# A New Transgenic Mouse Model for Studying the Neurotoxicity of Spermine Oxidase Dosage in the Response to Excitotoxic Injury

**DOI:** 10.1371/journal.pone.0064810

**Published:** 2013-06-19

**Authors:** Manuela Cervelli, Gabriella Bellavia, Marcello D'Amelio, Virve Cavallucci, Sandra Moreno, Joachim Berger, Roberta Nardacci, Manuela Marcoli, Guido Maura, Mauro Piacentini, Roberto Amendola, Francesco Cecconi, Paolo Mariottini

**Affiliations:** 1 Dipartimento di Biologia, Università “Roma Tre,” Rome, Italy; 2 Laboratory of Molecular Neuroembryology, Istituto di Ricovero e Cura a Carattere Scientifico (IRCCS) Fondazione Santa Lucia, Rome, Italy; 3 Faculty of Medicine, Nursing and Health Sciences, Monash University, Clayton, Australia; 4 Istituto Nazionale per le Malattie Infettive, IRCCS “L. Spallanzani,” Rome, Italy; 5 Dipartimento di Farmacia, Sez. Farmacologia e Tossicologia, Centro di Eccellenza per la Ricerca Biomedica CEBR, Università di Genova, Genoa, Italy; 6 Agenzia nazionale per le nuove tecnologie, l'energia e lo sviluppo economico sostenibile (ENEA), Il Centro Ricerche Casaccia, Sezione Tossicologia e Scienze Biomediche (BAS-BIOTECMED), Rome, Italy; Medical University Innsbruck, Austria

## Abstract

Spermine oxidase is a FAD-containing enzyme involved in polyamines catabolism, selectively oxidizing spermine to produce H_2_O_2_, spermidine, and 3-aminopropanal. Spermine oxidase is highly expressed in the mouse brain and plays a key role in regulating the levels of spermine, which is involved in protein synthesis, cell division and cell growth. Spermine is normally released by neurons at synaptic sites where it exerts a neuromodulatory function, by specifically interacting with different types of ion channels, and with ionotropic glutamate receptors. In order to get an insight into the neurobiological roles of spermine oxidase and spermine, we have deregulated spermine oxidase gene expression producing and characterizing the transgenic mouse model *JoSMOrec*, conditionally overexpressing the enzyme in the neocortex. We have investigated the effects of spermine oxidase overexpression in the mouse neocortex by transcript accumulation, immunohistochemical analysis, enzymatic assays and polyamine content in young and aged animals. Transgenic *JoSMOrec* mice showed in the neocortex a higher H_2_O_2_ production in respect to Wild-Type controls, indicating an increase of oxidative stress due to SMO overexpression. Moreover, the response of transgenic mice to excitotoxic brain injury, induced by kainic acid injection, was evaluated by analysing the behavioural phenotype, the immunodistribution of neural cell populations, and the ultrastructural features of neocortical neurons. Spermine oxidase overexpression and the consequently altered polyamine levels in the neocortex affects the cytoarchitecture in the adult and aging brain, as well as after neurotoxic insult. It resulted that the transgenic *JoSMOrec* mouse line is more sensitive to KA than Wild-Type mice, indicating an important role of spermine oxidase during excitotoxicity. These results provide novel evidences of the complex and critical functions carried out by spermine oxidase and spermine in the mammalian brain.

## Introduction

Putrescine (Put), spermidine (Spd), and spermine (Spm) are endogenous polyamines (PAs) essential for cell growth, proliferation, regeneration, and differentiation [1]–[4]. The functional role of natural PAs in the normal and diseased brain is under active research [5]–[9]. Early reports on the effects of PAs on neuronal firing and transmitter release were followed by compelling evidences showing that PAs are potentially involved in the regulation of a number of metabolic and electrophysiological processes [10]. Alteration of PAs content and their synthetic enzyme ornithine decarboxylase (ODC) in response to injuries, such as ischemia, hypoglycaemia, epilepsy, or trauma have been reported [11]–[15]. Even though these results suggest that PAs play an important role in neurodegeneration, the mechanisms whereby they participate in neuronal death, as well as the role of endogenous PAs in normal brain functioning, are to be elucidated yet. Specific interactions of PAs, in particular Spm, with different types of ion channels, have been reported [16], [17]. Intracellular PAs are able to block some types of K^+^ and Na^+^ channels and the glutamatergic AMPA (α-amino-3-hydroxy-5-methyl-4-isoxazolepropionic acid) and kainate receptors, while extracellular PAs modulate glutamatergic NMDA (N-methyl-D-aspartate) receptors [16], [18]–[21]. The catabolism of polyamines is finely regulated by the concerted action of three enzymes: spermidine/spermine-N1-acetyltransferase (SSAT), which acetylates Spm and Spd; acetyl-polyamine oxidase (APAO), which oxidizes these acetylated derivatives, regenerating Spd and Put, respectively; and the flavoprotein spermine oxidase (SMO), directly oxidizing Spm to produce Spd, 3-aminopropanal and hydrogen peroxide (H_2_O_2_). While APAO is constitutively expressed, SSAT and SMO are inducible enzymes, and have therefore been more extensively investigated [9]. Interestingly, it was shown that transgenic activation of PAs catabolism not only profoundly disturbs PAs homeostasis in most tissues, but also creates a complex phenotype affecting skin, female fertility, fat depots, pancreatic integrity and regenerative growth [22]. The SSAT overexpression in the Central Nervous System (CNS) resulted in significantly elevated threshold to pentylenetetrazol-induced seizure activity and protection against kainate-induced toxicity of transgenic animals [23]. Since SSAT overexpression resulted in even greater expansion of Put pool in different regions of the brain, a neuroprotective role of Put has been suggested [24]. Consistent with these data, also ODC overexpression, leading to Put accumulation, is neuroprotective [25]–[28]. In this scenario, we investigated the effects of SMO overexpression, so far unexplored, in a mouse genetic model. Since, among PA, Spm is the strongest modulator of GluRs and some types of K^+^ channels, we have generated a neocortex specific SMO overexpressing mouse model using a Cre/*loxP*-based recombination approach. This mouse model (named *JoSMOrec*) overexpresses SMO only in proneural populations of the hippocampus and the neocortex [29], allowing us to exclude any pleiotropic influence by other organs. In this work, we have studied the effect of SMO overexpression in the neocortex of young and aged mice, by analysing PA metabolism and glial stress markers expression. Aged SMO overexpressing mice show neuronal reduction, and light astrocyte and microglia activation in the cerebral cortex. To elucidate the possible role played by SMO in neuronal damage during excitotoxic insult, we have evaluated the effect of its overexpression after kainic acid (KA) systemic administration, known to induce epileptiform activity and excitotoxic mechanism activation in rodents [30]. Excitotoxicity refers to a process of neuronal death triggered by elevated levels of excitatory amino acid resulting in the opening of ionotropic glutamate receptors causing prolonged depolarization of neurons, the subsequent influx of calcium, and the activation of enzymatic and nuclear mechanisms of cell death [30]. In the *JoSMOrec* mouse model, we have also examined in the neocortex neuronal degeneration and astrocyte and microglia activation, analyzing the immunohistochemical expression of different cell markers, as well as H_2_O_2_ production. Moreover, in the attempt to characterize the neurodegenerative process occurring in SMO overexpressing KA-treated animals, we have performed an ultrastructural analysis of the injured neocortex. It resulted that the transgenic SMO mouse line is more sensitive to KA than Wild-Type (WT) mice. The results presented in this work provide novel evidences of the complex and crucial functions carried out by SMO and Spm in mammalian brain in physiological and pathological conditions.

## Materials and Methods

### Ethics statement

The experiments were carried out in accordance with the ethical guidelines for the conduct of animal research of the European Community's Council Directive 86/609/EEC. Formal approval of these experiments was obtained from the Italian Ministry of Health (Official Italian Regulation D.L.vo 116/92, “Communication to Ministero della Salute no. 70-VI/1.1”).

### Construction of plasmids and generation of transgenic mice

A *loxP*-*egfp*-polyA cassette was cloned into the *Eco*RI site of *Pcaggs* [31] followed by a *loxP*-(*Xho*I)-*IRES*-*lacZ*-polyA cassette resulting in *pJojo* vector. The coding sequence of *SMO* gene (GenBankTM accession number AY033889) was amplified with the primers SMO-1F and SMO2-R (Table 1) to introduce the *Xho*I restriction site. Amplified PCR product was restricted by *Xho*I and ligated with the restricted *Xho*I *pJojo* vector resulting in *pJoSMO* vector. The plasmids were used to generate *pJoSMO (GFP-SMO)* mice by pronuclear microinjection. Transgenic mice were identified by GFP fluorescence. Genomic PCR was performed with the primers SMO-3F and SMO-4R (Table 1). The transgenic mice were produced by the standard pronuclear microinjection technique [32]. Fertilized oocytes were obtained from superovulated BALB/c x DBA/2 mice mated with males of the same strain.

**Table 1:** Primers used in this study.

Target gene	Name	Primer sequence
*SMO*	SMO-1F	5′-TTTATACTCGAGCCTAGAAGGTGAGCACGGAC-3′
*SMO*	SMO-2R	5′-AAATATCTCGAGGGAACACATTTGGCAGTGAGG-3′
*SMO*	SMO-3F	5′-TCATCCCCTCGGGCTTCATG -3′
*SMO*	SMO-4R	5′-GGAACACATTTGGCAGTGAGG-3′
*SMO*	SMO-5F	5′-GTACCTGAAGGTGGAGAG-3′
*SMO*	SMO-6R	5′-TGCATGGGCGCTGTCTTGG-3′
*APAO*	APAO-1F	5′-GAGCCACCACTGCCTGCC-3′
*APAO*	APAO-2R	5′-CCATGTGTGGCTTCCCC-3′
*ODC*	ODC-1F	5′-TCCAGGTTCCCTGTAAGCAC-3′
*ODC*	ODC-2R	5′-CCAACTTTGCCTTTGGATGT-3′
*SSAT*	SSAT-1F	5′-CGTCCAGCCACTGCCTCTG-3′
*SSAT*	SSAT-2R	5′-GCAAGTACTCTTTGTCAATCTTG-3′
*rpS7*	rpS7-1F	5′-CGAAGTTGGTCGG -3′
*rpS7*	rpS7-2R	5′-GGGAATTCAAAATTAACATCC -3′
*β-actin*	β-actin-1F	5′-TGTTACCAACTGGGACGACA-3′
*β-actin*	β-actin-2R	5′-AAGGAAGGCTGGAAAAGAGC-3′

### Animals and kainate administration

All double transgenic animals *JoSMOrec* were obtained from a cross between *JoSMO* (BALB/cx DBA/2 as described above) and *Dachshund-Cre* (DBA/2) mice. *Dachshund-Cre* mouse line expresses Cre in proneural population of the nervous system [29] and leads to the recombination in the crossed progeny to produce JoSMOrec mice. Animals were housed under controlled temperature (20±1°C), humidity (55±10%), and on a 12-h light/dark schedule. Food and water were provided *ad libitum*. All experiments were performed on independent groups of mice. Kainic acid was dissolved in isotonic saline solution (50 mM NaPi pH 7.2, 100 mM NaCl) and administered subcutaneously at a dose of 25 mg/kg p.c. Following KA administration, mice were monitored continuously for 3–4 h for the onset and extent of seizure activity. Seizures were rated according to a previously defined scale by Schauwecker [30]: stage 1, immobility; stage 2, forelimb and/or tail extension, rigid posture; stage 3, repetitive movements, head bobbing; stage 4, rearing and falling; stage 5, continuous rearing and falling; stage 6 severe tonic-clonic seizures.

### *In situ* detection of H_2_O_2_ in mouse neocortex and cerebellum

*In situ* detection of H_2_O_2_ in mouse neocortex and cerebellum was carried out by exploiting the fluorogenic peroxidase substrate AUR (Amplex UltraRed reagent, Invitrogen) that reacts in a 1∶1 stoichiometry with H_2_O_2_ to produce a highly fluorescent reaction product (excitation/emission maxima approximately 568/581 nm). Neocortex and cerebellum samples from Tg and Sg mice were stained by incubation with 0.1 mM AUR, for 5 min under vacuum (−400 mbar). After washing cortex and cerebellum we observed under LSCM (HeNe laser emitting at wavelength of 543 nm). The selected emission bands ranged from 550 to 700 nm. The selected bands do not overlap with the excitation and emission wavelength from GFP (488 and 509 nm, respectively) protein expressed by Tg and Sg mice.

### RT-PCR analysis

The relative levels of SMO, APAO, ODC, SSAT, β-actin and rpS7 transcripts were measured by RT-PCR with specific primers listed in Table 1. Total RNA was isolated from brain cortex and cerebellum as control by TRIZOL reagent (Gibco BRL), according to the manufacturer's instructions. Synthesis of the cDNAs from the RNAs of different mouse organs were performed by primer random examers in 20 µl reaction volume containing 1 µg of total RNA, according to the manufacturer's instructions (SuperScriptIII First-Strand Synthesis System for RT-PCR, Invitrogen). Aliquots of reverse-transcribed-RNA were amplified within with Taq DNA polymerase (M-Medical) in the linear range and in saturating experimental conditions by 20, 25, 30 or 35 PCR cycles: denaturation at 94°C for 1 min, annealing at 60°C for 30 sec and extension at 72°C for 1 min. The RT-PCRs were normalized by the comparison of the β-actin and rpS7 controls. Further control reaction mixtures, either without template (not shown) or RT enzyme (not shown), were uniformly negative.

### Determination of SMO and APAO enzyme activity and PA content

Polyamine oxidase activity of SMO/APAO was assayed using a modification of the chemiluminesence analysis reported by Wang et al. [33]. Briefly, luminol-dependent chemiluminescence was determined using a Lumat LB 9507 G&G BERTHOLD luminometer. Luminol was prepared as a 100 mM stock solution in DMSO and diluted to 100 µM with H_2_O, immediately prior to use. Tissue cortex and cerebellum extract was assayed in a 83 mM glycine buffer pH 8.3, 20 µg/ml horseradish peroxidase, 0.2 mM 2-bromoethylamine (catalase inhibitor), 15 µM deprenyl (copper-containing amine oxidase inhibitor), 0.15 µM clorgyline (mitochondrial oxidase inhibitor), and 500 µM Spm or 500 µM *N* ^1^-acetylSpm as substrate, to determine SMO or APAO activity respectively. All reagents, with the exception of substrate, were combined and incubated for 5 minutes at 37°C, then 5 nmol luminal was added and incubated again at 37°C for 2 minutes, transferred to the luminometer where spermine or *N*1-acetylspermine was added, and the resulting chemiluminescence was integrated over 40 seconds. Polyamine concentration was determined as described in Mates et al. [34].

### Light and electron microscopy

Transgenic (Tg) and syngenic (Sg) mice were sacrificed 1 and 3 days after KA administration, or 1 day after vehicle injection. Animals were transcardially perfused at room temperature (RT) with 0.1 M phosphate buffer (PB), pH 7.3, followed by 4% freshly depolymerised paraformaldehyde in PB. Brains were removed 1 h after perfusion and sagittaly cut along the midline. The right halves of the brains were processed for immunohistochemical studies, while the left halves were collected for morphological analyses at the light and electron microscopic level.

#### Immunohistochemistry

Samples were dehydrated in graded ethanol, transferred to Bioclear (BioOptica, Milan, Italy), then to a 1∶1 mixture of Bioclear and paraffin, and finally embedded in paraffin. Sagittal, 5 µm thick sections were then serially cut by a microtome and collected on Vectabond (Vector, Burlingame, CA, USA) pre-coated slides. Sagittal, 100 µm thick sections were obtained by a vibratome, and collected in phosphate buffer. Serial, sagittal brain sections from WT, Sg and Tg animals were deparaffinized by using xylene and graded ethanol and rehydrated. Slides were then immersed in 10 mM sodium citrate buffer, pH 6.1, and processed for the antigen-retrieval procedure, using a microwave oven operated at 720W for 10 min [35]. After cooling, slides were transferred to phosphate buffer saline (PBS) containing 5% (w/v) non-fat dry milk, for 1 h at RT, then incubated for 48 h at 4°C with either of the following antibodies, diluted in PBS containing 2.5% (w/v) non-fat dry milk: 1∶100 AntiSMO (Proteintech Group), 1∶500 NeuN (Chemicon), 1∶500 GFAP (Dako Cytomation) and 1∶500 Iba1 (Biocare Medical). In control sections, the primary antibody was omitted or substituted with normal rabbit serum. Slides were then incubated for 1 h at RT with biotinylated goat anti-rabbit IgG or goat anti-mouse IgG (Vector), diluted 1∶200 in PBS containing 1% normal goat serum (Vector). Immuno-complexes were revealed by means of an avidin biotin system (Vectastain Elite ABC kit, Vector), using 3,3′-diamino-benzidine (DAB Substrate kit for Peroxidase, Vector), as the chromogen. Slides were finally dehydrated and mounted with Eukitt (Kindler GmbH & Co., Freiburg, Germany). Sections were observed under an Olympus BX 51 microscope, equipped with a Leica DFC 420 camera; electronic images were captured by a Leica Application Suite system, and composedin an Adobe Photoshop CS2 format.

#### Morphological analysis

After brain dissection, small pieces from the left neocortices of Sg and Tg mice were postfixed in 1% OsO_4_ in PB, dehydrated, and embedded in epoxy resin. Specimens were cut on a Leica Reichert Supernova ultramicrotome. Semithin sections were stained with toluidine blue and observed in a LEITZ DMRB light microscope. Ultrathin sections were briefly contrasted with uranyl acetate and examined by a Zeiss CM 900 electron microscope. Images of semithin and ultrathin sections were electronically captured and composed in an Adobe Photoshop CS3 format.

### Statistical analysis

Cell counting in each field (number of cells/0.24 mm^2^) after immunohistochemical analyses were performed on mouse cortex slides (n = 6) from six independent individuals for each groups; data are presented as mean ± S.D. Data significance was assessed by one or two-way ANOVA tests. In particular, one-way analysis of variance and post-hoc test Bonferroni has been used for comparing several groups, while two-way analysis of variance and post-hoc test Bonferroni has been used for comparing different groups and treatment effects. Probabilities of p<0.05 = *, p<0.01 = ** and p<0.001 = *** were taken as levels of significance. Toluidine blue stained semithin sections from mouse neocortices of Sg (n = 6) and Tg mice (n = 6) were examined using a LEITZ DMRB light microscope, at low-to-high magnification. We evaluated the percentage of dying neurons *vs.* the total cell number, considering as altered those neurons showing high condensation and strong nuclear basophilia. Three different embedding blocks were analyzed for each condition, and a minimum of 200 cells per block were observed. Cell counting was performed on slides from three independent individuals; data are presented as mean ± S.D.

## Results

### Conditional activation of *SMO in vivo*

**Figure 1:**
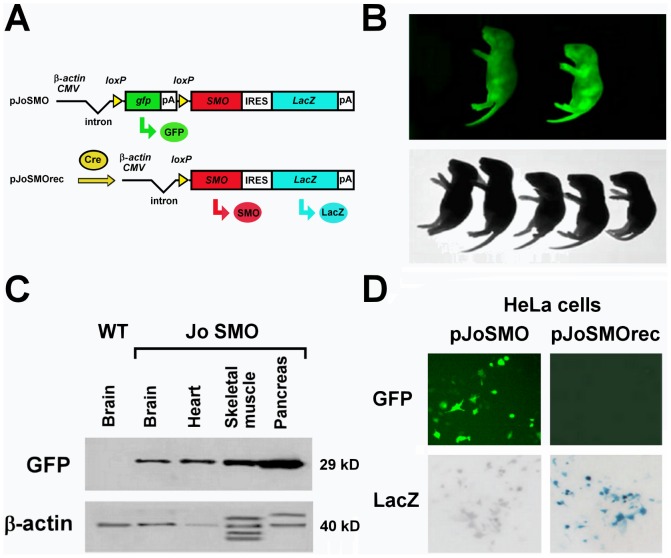
*JoSMO* mouse line generation. **A**. Scheme of pJoSMO and, upon Cre recombination, of pJoSMOrec plasmids. The β-actin/CMV fusion promoter drives the ubiquitous expression of the *gfp* (green arrow, Green Fluorescent Protein) reporter gene. Upon Cre recombination the *gfp*-stop cassette is excised, leading to simultaneous expression of *SMO* (red arrow) and of the second reporter gene, *lacZ* (blue arrow, β-galactosidase), via an IRES sequence. **B**. *JoSMO* mice exhibited widespread GFP fluorescence. **C**. Western blot analysis confirms the presence of GFP in different organs. **D**. The Cre recombination was analysed with pJoSMO and pJoSMOrec plasmids transfection in HeLa cells. After transfection, GFP fluorescence and LacZ staining on HeLa cells were monitored.

**Figure 2:**
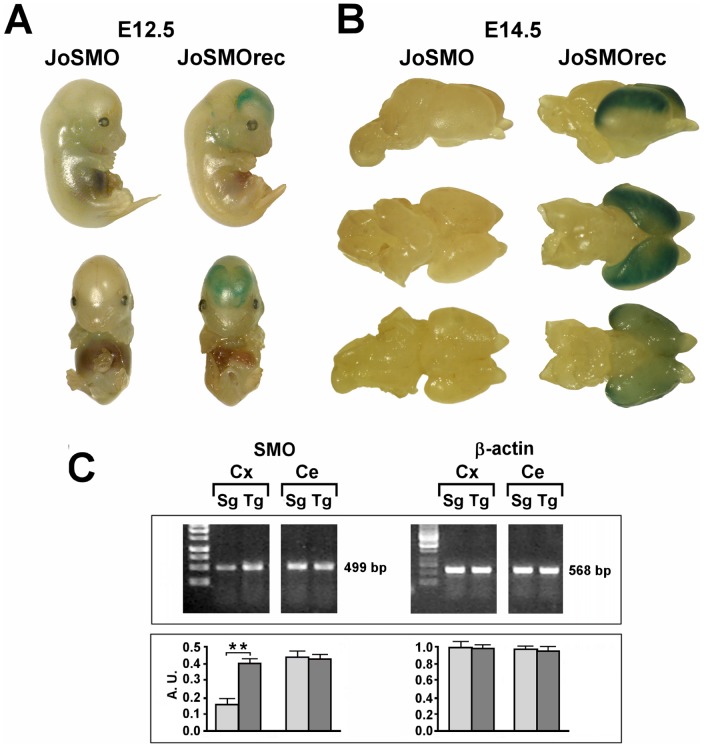
Functional analysis of the second reporter gene LacZ of whole-mount and isolated brains. **A**. LacZ staining of whole-mount at E12.5 mouse developmental stage. **B**. LacZ staining of brain from *JoSMOrec* mice at E14.5 developmental stage. **C**. SMO transcript level analyses by semiquantitative RT-PCR in the neocortex of *JoSMOrec* mice and densitometric. Representative RT-PCR experiments from three independent replicas are shown. Densitometric analyses of PCR gel bands, represent the measurements done on three separate experiments. The β-actin gene expression was used for normalization. An arbitrary densitometric unit bar graph (A.U.) is shown. The p values were measured with the one-way ANOVA test and post-hoc test Bonferroni (**, p<0.01). Sg, syngenic mice; Tg, transgenic mice; Cx, cortex; Ce, cerebellum.

For conditional activation of *SMO* we generated a construct (*pJoSMO*) that contains a floxed *gfp*-stop cassette under control of the β-actin/CMV fusion promoter [31], driving ubiquitous expression of the *gfp* (Green Fluorescent Protein) reporter gene. Upon Cre recombination the *gfp*-stop cassette is excised, leading to simultaneous expression of *SMO* and of the second reporter gene, *lacZ* (β-galactosidase), via an IRES sequence (Figure 1A). The transgenic mouse line generated with this construct was named *JoSMO* and the characterization of the mice was carried out to select the founders possessing a single copy inserted transgene by Southern blot analysis (not shown) and overexpressing GFP in all tissues (not shown). After genotyping, the selected *JoSMO* mice exhibited widespread GFP fluorescence and Western blot analysis confirm the presence of GFP in different organs (Figure 1B–C). In order to test the recombination of the integrated construct, *JoSMO* were crossed with *Dachshund-Cre* mice expressing Cre [29] and directing recombination in proneural population in the nervous system. This cross produced a double transgenic mouse line hereafter named *JoSMOrec* (or Tg for the sake of simplicity), while single transgenic *JoSMO* mice, coming from the same offspring, were used as control and referred as syngenic animals (Sg). Before making the cross *JoSMO;Dachshund-Cre*, the Cre recombination was monitored with pJoSMO and pJoSMOrec plasmids transfected in HeLa cells. After transfection, GFP fluorescence and LacZ staining on HeLa cells were monitored (Figure 1D), and PCR was performed, using the primers JoP6F and JoP6R which bind 5′ and 3′ ends of the floxed *gfp*-stop cassette, the genetic constructs were confirmed (not shown). Expression of the second reporter LacZ was tested by staining of whole-mount and isolated brains (Figure 2A–B). The brain of *JoSMOrec* exhibited LacZ specifically in the cerebral cortex at E12.5 and E14.5 mouse developmental stages (Figure 2A–B). The SMO overexpression in the neocortex of these mice was assessed by semiquantitative RT-PCR, which revealed an approximate two-fold increase of SMO transcript level (Figure 2C–D).

### Immunohistochemical analysis of *JoSMOrec* neocortex

**Figure 3:**
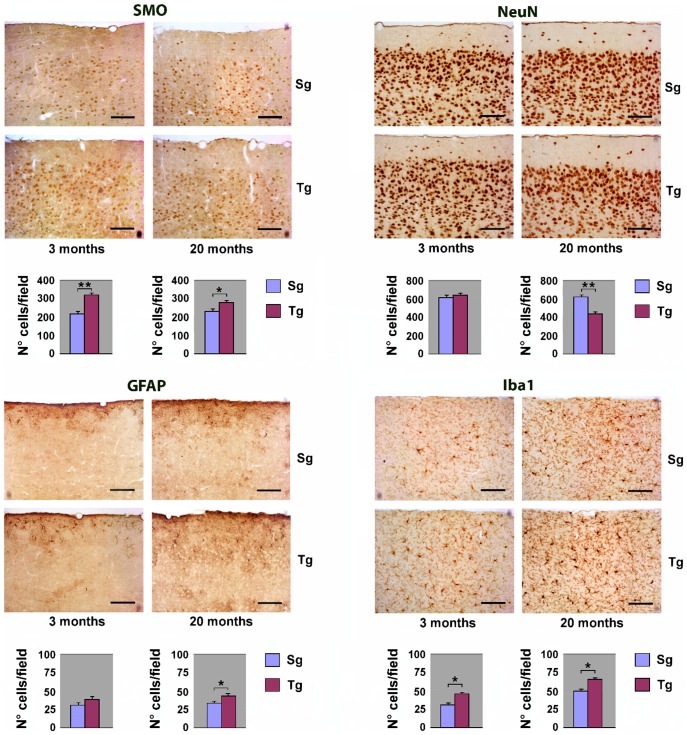
Immunohystochemical analysis of neocortex from Tg and Sg mice. Sagittal brain slices from *JoSMOrec* mice were stained with antibodies directed against SMO, NeuN, GFAP and Iba1. Slides of neocortex from 3 and 20 months old mice were analyzed. Cell counting is expressed as number of positive cells per 0.24 mm^2^ area. The p values were measured with the one-way ANOVA test and post-hoc test Bonferroni (*, p<0.05; **, p<0.01; ***, p<0.001). Sg, syngenic mice; Tg, transgenic mice.

To study the overall cytoarchitecture of Tg mouse neocortex, as compared to its Sg counterpart, we analysed the immunohistochemical distribution of SMO and of specific neural cell markers. Serial sagittal paraffin sections from Tg mice were immunostained using antibodies to SMO, to the neuronal marker NeuN (neuronal nuclei), to the astroglial marker GFAP (glial fibrillary acid protein), and to the microglial marker Iba1 (ionized calcium binding adapter molecule). SMO immunoreactivity was sensibly enhanced in both young and old Tg mice, compared to controls. Quantitative evaluation of immunohistochemical data, indicates increases of about 50% and ∼15% in the number of positive cells in Tg young and old neocortex, with respect to Sg (Figure 3). As to the general cortical cytoarchitecture of Tg and Sg mice, we did not detect any difference in the number of NeuN-positive neurons, between Tg and Sg young mice. By contrast, a significant reduction of neurons was observed in old Tg mice (∼30% lower than Sg) (Figure 3). Using an anti-GFAP antibody, we could observe in old Tg mouse neocortex a numerical increase of astrocytes, which also appear hypertrophic and highly ramified (Figure 3). We also observed a significant increase of Iba1-stained cells in the aged neocortex, indicating a microglial activation. Interestingly, the neocortex of both young and old Tg mice showed more intense Iba1-immunoreactivity, when compared to Sg mice (Figure 3). As an additional control, the immunohistochemical analysis on neocortex with the same panel of antibodies used in Figure 3 was performed also on non-transgenic littermates (WT) (Figure S1).

### SMO enzymatic activity and PA content in the neocortex of young and old *JoSMOrec* mice

**Figure 4:**
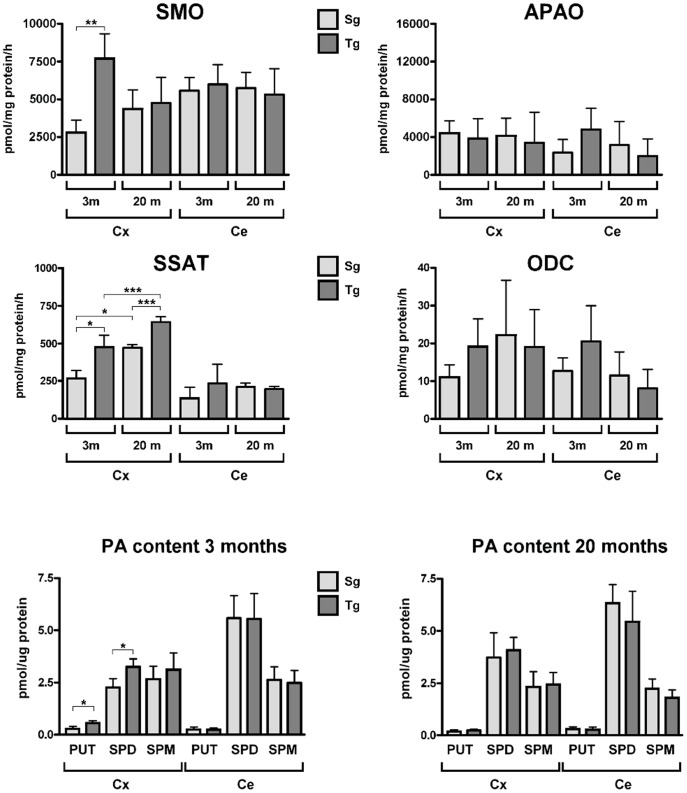
SMO, APAO, ODC and SSAT activities and PA content of Tg and Sg mice. Enzyme activities of SMO, APAO, ODC and SSAT and PA content from cortex and cerebellum of 3 (3 m) and 20 (20 m) months old mice were analyzed. The p values were measured with one-way ANOVA test and post-hoc test Bonferroni (*, p<0.05; **, p<0.01). Sg, syngenic mice; Tg, transgenic mice; Cx, cortex; Ce, cerebellum.

SMO enzyme activity was measured in homogenates from the neocortex and cerebellum (chosen as a reference brain area) of young and old *JoSMOrec* mice. We observed a 3-fold-increase in the neocortex of Tg 3-month-old mice respect to aged-matched Sg, while no difference was detected between Tg and Sg mice at 20 month of age (Figure 4). In parallel, the activity of the most representative enzymes of PA metabolism, namely APAO, SSAT and ODC, were assayed. While no differences were observed in APAO and ODC enzymatic activities, SSAT showed significantly increased activity in the neocortex of both young and aged SMO transgenic mice, especially in 3-month-old animals (100% *vs.* a 20% increase, in young and old mice, respectively). As to PA content, only Put and Spd resulted to be slightly increased in young Tg mice (Figure 4).

### *In situ* detection of H_2_O_2_ in the neocortex of *JoSMOrec* mice

**Figure 5:**
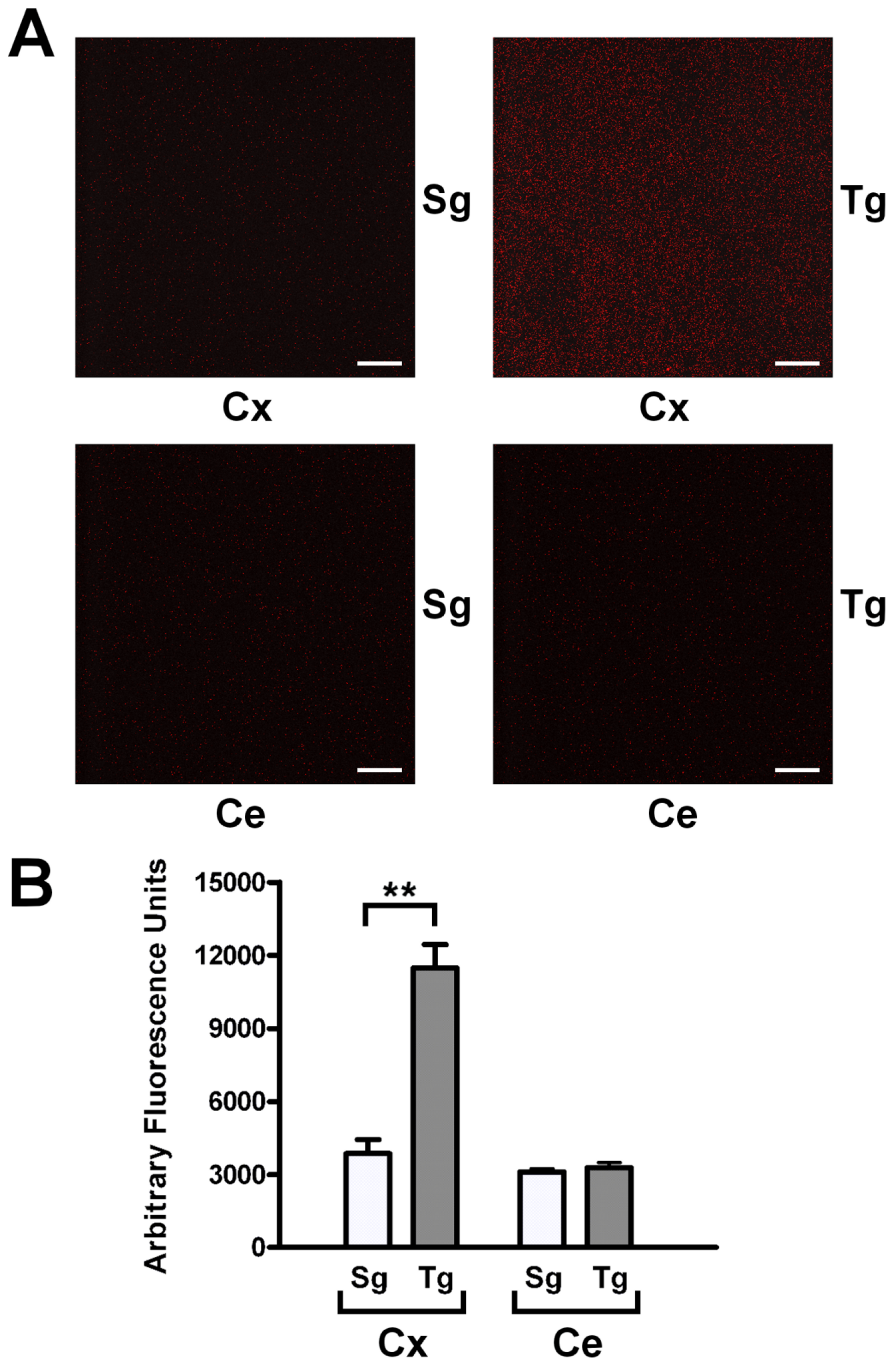
*In situ* detection of H_2_O_2_ in mouse neocortex and cerebellum. A. Mouse neocortex and cerebellum from Tg and Sg were stained with AUR reagent and analyzed by LSCM (HeNe laser emitting at wavelength of 543 nm). B. Quantification of stained H_2_O_2_ molecules. The p values were measured with the one-way ANOVA test and post-hoc test Bonferroni (**, p<0.01). Sg, syngenic mice; Tg, transgenic mice; Cx, cortex; Ce, cerebellum.

The production of H_2_O_2_ of the neocortex of *JoSMOrec* mice have been evaluated by *in situ* AUR staining (Figure 5). Neocortex from Tg mice showed a three-fold increase of H_2_O_2_ production compared to Sg controls, no difference of staining was observed in the cerebellum from Tg and Sg mice (Figure 5A). Quantification of stained H_2_O_2_ molecules was plotted with a histogram shown in Figure 5B.

### Excitotoxic conditions on *JoSMOrec* mice induced by kainate treatment and behavioural phenotype evaluation

Kainic acid (2-carboxy-4-isopropenylpyrrolidin-3-ylacetic acid, KA) is an acidic pyrolidine isolated from the seaweed *Digenea simplex*, and is the most potent of the common exogenous excitotoxins [36]–[38]. Its neurotoxic threshold is nearly two orders of magnitude lower than that of the other receptor-specific agonists, namely, N-methyl-D-aspartic acid and quisqualic acid [36]. To investigate the possible involvement of SMO in excitotoxicity, we administered KA at dose of 25 mg/Kg [39], to 3-month-old Tg and Sg mice whereas control animals were treated with saline solution (vehicle). After KA treatment, animals were monitored for 6 h to assess the onset time and the level of seizures activity, which was scored according to Schauwecker [30]. Behavioural response is represented in Table 2. Most of the Sg mice (∼63%) displayed a milder phenotype (stage 1 to 3), than Tg mice. By contrast, within 30 min of injection 58% of Tg mice showed progressive seizures, ranging from stage 4 to 5, while over the next hour about 35% of animals even displayed stage 6. Overall, these data point out a higher sensitivity to KA of Tg mice, compared to their Sg counterpart. No seizure activity was observed in Tg and Sg animals treated with vehicle. After 6 h monitoring, all mice recovered a normal behaviour and were kept up to 1 or 3 days before being processed.

**Table 2:** Behavioural evaluation following KA treatment.

Mouse line	Stage 1	Stage 2	Stage 3	Stage 4	Stage 5	Stage 6
Tg %	5.38	3.85	23.08	15.38	7.69	34.62
Sg %	33.33	7.41	22.22	14.81	3.70	18.52

Scored mice are expressed as percentage. Tg, transgenic; Sg, syngenic.

### Morphological analysis of Sg and Tg neocortex after KA treatment

**Figure 6:**
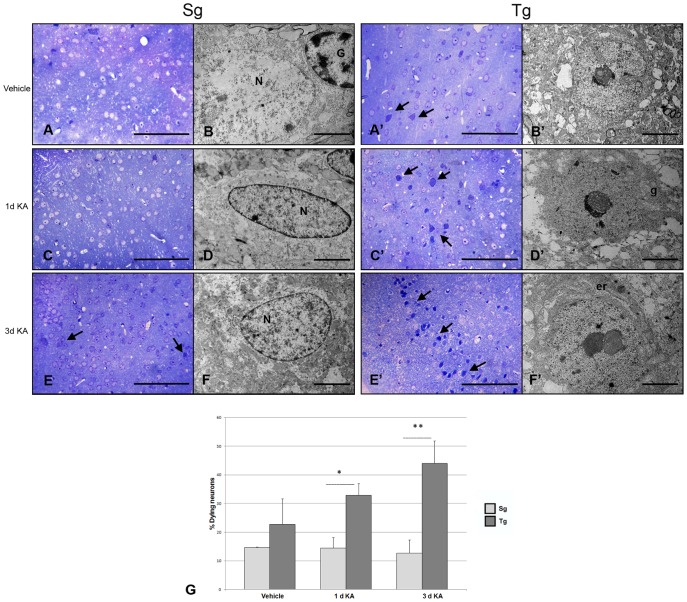
Light and electron microscopic analysis of Tg and Sg neocortex. **A, C, E** semithin and ultrathin **B, D, F** sections from Sg neocortex, showing cell morphology in all the examined conditions. Condensed neurons are marked by black arrows. At ultrastructural level, neurons show regular nuclei (N) and preserved cytoplasmic organelles. G, glial cells. Scale bars: 1 mm (**A, C, E**), and 5 µm (**B, D, F**). **A′, C′, E′** semithin and ultrathin **B′, D′, F′** sections from Tg neocortex. Neurons with abnormal morphological features, including cytoplasmic condensation and strong nuclear basophilia are marked by black arrows. The electron microscopic analysis of damaged neurons reveals irregular nuclear envelope, heterochromatin clumps, and cytoplasmic shrinkage (**D′** and **F′**). These alterations are accompanied by enlargement of Golgi apparatus (g) and endoplasmic reticulum cisternae (er). The graph shows the quantification of dying neurons in Tg and Sg neocortices at 1 day after vehicle injection, at 1 and 3 days after KA injection. The p values were measured with the two-way ANOVA test and post-hoc test Bonferroni (*, p<0.05; **, p<0.01; ***, p<0.001). Scale bars: 1 mm (**A′, C′, E′**), and 5 µm (**B′, D′, F′**). Sg, syngenic mice; Tg, transgenic mice.

Neocortical samples from Tg and Sg mice injected with KA/vehicle were analyzed 1 and 3 days after treatment. Toluidine blue stained semithin sections were utilized to assess the extent of tissue damage caused by the toxic agent (Figure 6A, C, E, A′, C′, E′). The Tg brain resulted especially susceptible to KA induced damage, in that several neocortical neurons showed abnormal morphological features, including cytoplasmic condensation and strong nuclear basophilia (arrows in Figure 6E, A′, C′, E′). At the ultrastructural level, Tg neurons appeared highly electron dense in their cytoplasmic and nuclear compartments, the latter showing heterochromatin clumps and nuclear envelope invaginations (Figure 6B′, D′, F′). Quantification of dying neurons in semithin sections demonstrated a significantly higher number in KA treated Tg neocortices, compared to their Sg counterparts. Specifically, 1 day after KA injection, we detected a more than two-fold increase in the number of condensed neurons, while 3 days after injection an approximate three-fold increase was observed (Figure 6G).

### Immunohistochemical analysis of KA-treated *JoSMOrec* neocortex

**Figure 7:**
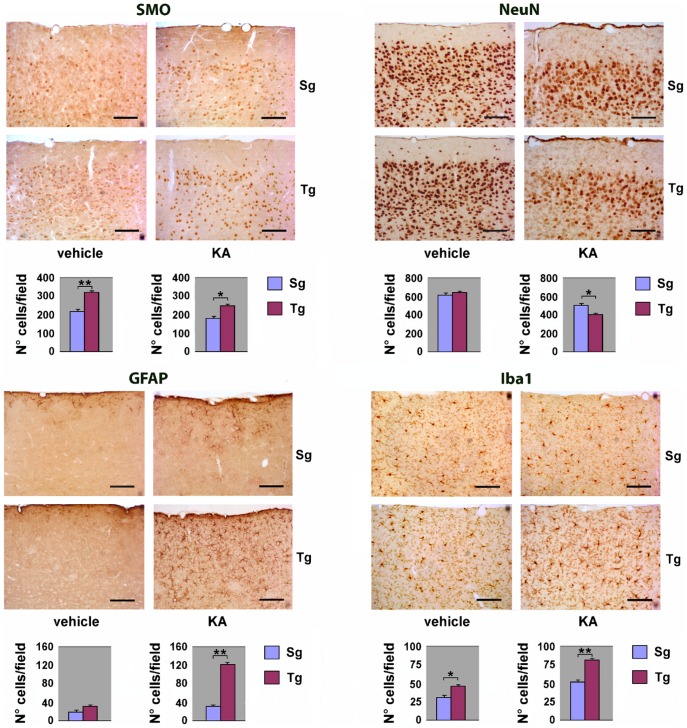
Immunohystochemical analysis of neocortex from KA treated Tg and Sg mice. Sagittal brain slices from *JoSMOrec* mice were stained with antibodies directed against SMO, NeuN, GFAP and Iba1. Slides of neocortex from KA treated 3 months old mice were analyzed. Cell counting is expressed as number of positive cells per 0.24 mm^2^ area. The p values were measured with the two-way ANOVA test and post-hoc test Bonferroni (*, p<0.05; **, p<0.01). Sg, syngenic mice; Tg, transgenic mice.

Immunohistochemical analysis using antibodies against SMO, NeuN, GFAP and Iba1 was performed on serial sagittal paraffin sections of the neocortex from Tg and Sg mice treated with vehicle or KA, and sacrificed 1 or 3 days after treatment. The Tg neocortex showed a higher SMO immunoreactivity in terms of both intensity and number of positive cells (∼30% increase), compared to Sg one (Figure 7). After KA injection SMO immunoreactive cells decreased in both Tg and Sg animals, compared to sham animals. Nevertheless, Tg injured mice displayed a higher number of positive cells than Sg treated animals (∼20% increase). Using NeuN antibodies, we could not observe any difference in the number of neurons between Tg and Sg mice treated with vehicle, while a reduction (∼20%) was detected in KA injected Tg mice (Figure 7), indicating a higher loss of neurons in Tg mice. Since KA mediated lesions induce astrogliosis, we examined GFAP immunodistribution in the neocortex from KA/vehicle injected mice. Sham animals showed little GFAP staining with no significant differences between Sg and Tg mice, whilst KA treatment resulted in intense GFAP reactivity, especially dramatic in Tg neocortex. In these samples, astrocytes are not only increased in number (around 200% compared to sham), but show morphological features, including hypertrophy and wide ramification, typical of reactive astrogliosis. Consistently, the microglial marker Iba1, which is upregulated during activation of these cells, was highly expressed in the neocortex of Tg mice. Indeed, vehicle-injected Tg mice also showed a higher number (40% increase) of Iba1-positive cells, compared to Sg mice. After KA treatment this increase reached 60%, indicating a more dramatic microgliosis occurring in Tg than in Sg mice (Figure 7). Altogether, immunohistochemical results, demonstrating higher neuronal loss and a stronger astroglial and microglial activation in Tg mice compared to Sg mice, support the hypothesis that *JoSMOrec* mice are especially sensitive to KA excitotoxicity.

### Activity assays of PA key metabolic enzymes

**Figure 8:**
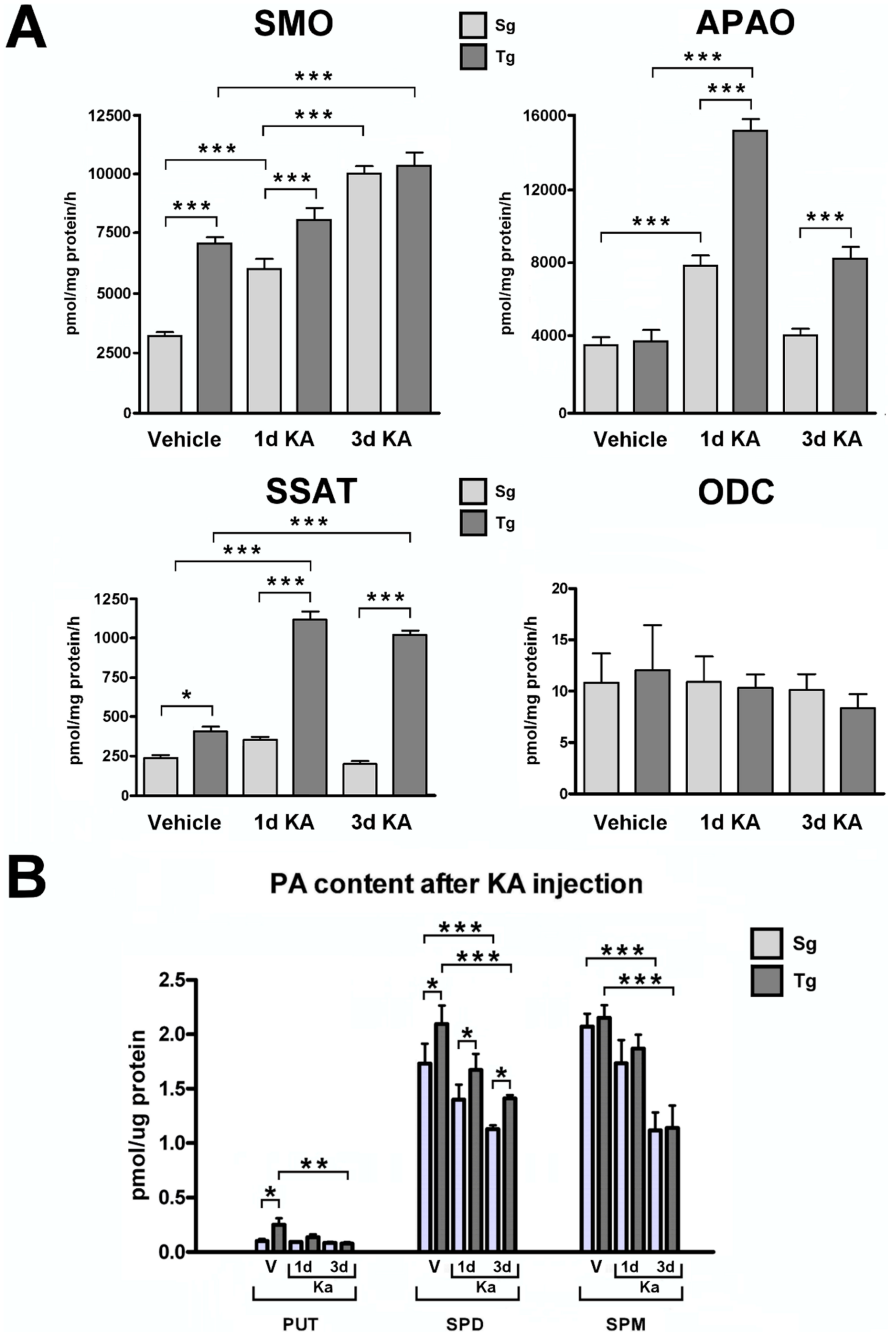
SMO, APAO, ODC and SSAT activities and PA content of KA treated Tg and Sg mice. Enzyme activities of SMO, APAO, ODC and SSAT and PA content from neocortex of 3 months old mice were analyzed after 1 and 3 days of KA treatment. The p values were measured with the two-way ANOVA test and post-hoc test Bonferroni (*, p<0.05; **, p<0.01; ***, p<0.001). Sg, syngenic mice; Tg, transgenic mice.

In order to investigate PA metabolism in KA-injected mice, SMO, APAO, SSAT and ODC enzymatic activities were analyzed in Tg and Sg neocortex. To this purpose, animals were sacrificed 1 or 3 days after KA/vehicle treatment. Both Tg and Sg mice treated with saline solution displayed SMO, APAO, SSAT and ODC activities comparable with the ones of untreated animals (Figure 8; compare with Figure 4), demonstrating that vehicle injection does not affect any of the above enzymatic activities (see below). KA treatment induced an increase of SMO enzymatic activity in both Tg and Sg neocortices at 1 and 3 days after injection. The sample taken 1 day after injury showed a 20% increase of SMO activity in Tg mice, while no difference was observed between Tg and Sg mice at 3 days (Figure 8A). While no difference was observed in APAO basal activity between Tg and Sg neocortices, 1 day after injury this enzyme showed a strong increase in both genotypes, especially dramatic in the Tg neocortex (300%). Furthermore, three days after KA injection, APAO activity decreased in both Tg and Sg samples, down to the basal level in Sg mice, but still higher by two folds than the basal level in Tg mice. KA treatment resulted in a significant increase by 200% of the SSAT activity at 1 day and remained significantly higher at 3 days in the Tg mice respect to Sg mice (Figure 8A). ODC activity measured at 1 and 3 days after KA injury, was apparently unaffected in either Tg or Sg mice (Figure 8A).

### PA content analysis

To examine possible changes in PA content following KA treatment, Put, Spd and Spm levels in the neocortex were measured by HPLC (Figure 8B). Put level was higher in Tg mice compared to Sg mice in vehicle injected animals, while KA treatment leads to its decrease in Tg mice at 1 and 3 days. At difference Put levels in Sg mice were unchanged. As regards Spd level, it was higher in all Tg mice (control and KA treated animals) compared to Sg mice. But, a general decrease of Spd content was observed following the treatment in either Sg or Tg mice, particularly after 3 days. No modifications in Spm level were found between Sg and Tg mice in both sham and treated animals. However, KA administration induced a significant reduction of Spm content in Sg and Tg mice after 3 days of treatment.

### Transcript accumulation of PA key metabolic genes

**Figure 9:**
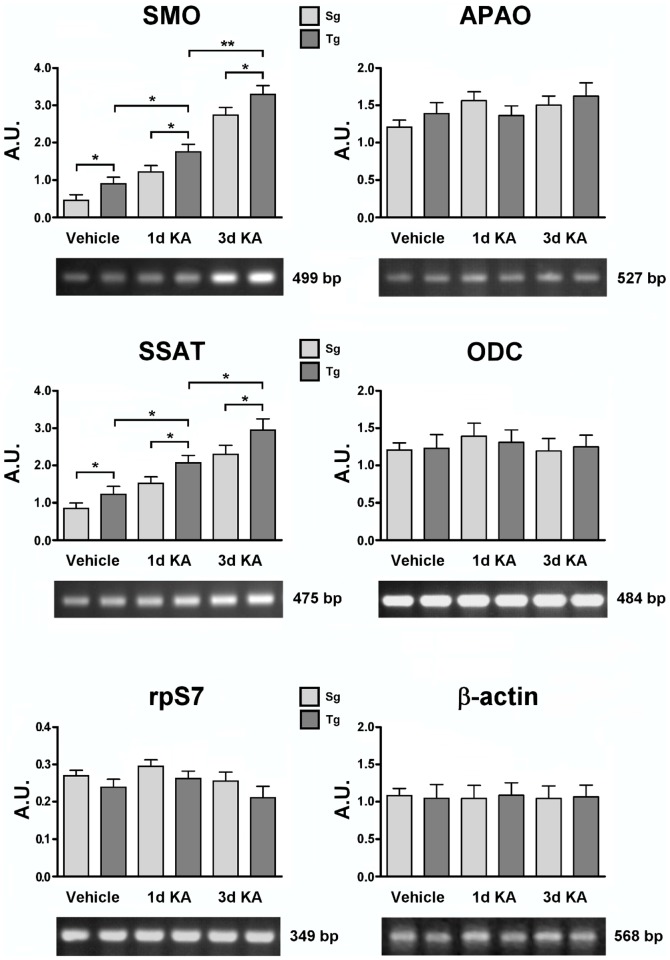
SMO, APAO, ODC and SSAT transcript level analyses of KA treated Tg and Sg mice. SMO, APAO, ODC and SSAT transcript accumulation of 3 months old mice were analyzed after 1 and 3 days of KA treatment. Representative RT-PCR experiments from three independent replicas are shown. Densitometric analyses of PCR gel bands, represent the measurements done on three separate experiments. The β-actin and/or rpS7 gene expression was used for normalization. An arbitrary densitometric unit bar graph (A.U.) is shown. The p values were measured with the two-way ANOVA test and post-hoc test Bonferroni (*, p<0.05; **, p<0.01). Sg, syngenic mice; Tg, transgenic mice.

Levels of SMO, APAO, ODC and SSAT mRNAs were examined in neocortical samples from Tg and Sg mice injected with KA/vehicle. The housekeeping control β-actin protein and ribosomal protein S7 (rpS7), were also probed to quantify the amplified samples [40]. Figure 9 shows the transcripts accumulation of *SMO* gene in Tg mice, which is significantly higher than Sg mice in basal conditions, and increases after KA treatment. By contrast, no significant differences were observed in either APAO or ODC transcript accumulation in Tg and Sg mice injected with KA or vehicle (Figure 9). Regarding SSAT transcript accumulation, we observed gene induction after injury in both Tg and Sg neocortex, furthermore SSAT mRNA levels resulted higher in Tg than in Sg samples (Figure 9).

## Discussion and Conclusions

In the last decades, considerable interest has been devoted to understand the possible role of PAs as modulators of several types of ion channels [16], [41]. Studies on transgenic animals with modified PA metabolism have also contributed to elucidating the involvement of PAs in brain functioning and pathology. Several genes encoding enzymes of PA metabolic pathways have so far been targeted by genetic engineering, and transgenic models have been used to address the issue of whether altered PA metabolism in response to brain injury is a cause of neuronal damage or a sign of plasticity and neuroprotection. Indeed, enhanced Put accumulation with marginal changes in the Spd and Spm content is linked to a vast majority of neurotoxic insults, either chemical or physical, in different brain regions, usually through ODC induction [10], but occasionally also as a result of SSAT induction [42]. Examination of transgenic animals with life-long overexpression of ODC and enhanced brain accumulation of Put failed to reveal any signs of neuronal degeneration until the age of two years [43]. These animals were also protected from physically or chemically induced seizure activity, while showing impaired spatial learning and memory [44]. A number of studies performed on transgenic mice and rats have indicated that overexpression of ODC and grossly elevated Put brain levels, partially protects against ischemia-reperfusion injury [25]–[28]. Similarly, transgenic mice with greatly expanded brain Put pools resulting from SSAT overexpression were relatively less sensitive to kainate-induced general and neuronal toxicity [45] and showed elevated threshold to pentylentetrazol-induced convulsions, in comparison with wild-type animals [23]. Interestingly, the latter difference was not detected when the convulsant was administered concomitantly with ifenprodil, a NMDA antagonist [23]. On the other hand, SSAT overexpressing mice are hypomotoric and less aggressive than wild-type animals and show impaired spatial learning [46]. Spermidine and Spm are agonists of NMDA receptor [47], [48], while Put is believed to acts as a weak antagonist for this receptor with questionable physiological significance [48]. Functional NMDA receptors are needed for synaptic plasticity and spatial learning [49], [50], but prolonged activation of the receptor is associated with neuronal damage. Taken together the results obtained with transgenic models indicate that the strikingly expanded brain Put pools in the transgenic animals create a partial blockade of NMDA receptors [24]. In order to highlight the role played by the SMO enzyme in normal brain functioning and during neurodegenerative processes we have genetically engineered a mouse model overexpressing *SMO* gene in the cerebral cortex. This genetic model is conceptually new and makes it possible to express SMO conditionally, in particular, a transgenic mouse founder has been created (*JoSMO*), which ubiquitously expresses GFP at a high level, but does not overexpress SMO. When this model is bred with another transgenic line expressing Cre recombinase in a tissue specific way, SMO overexpression takes place in this specific tissue. This strategy has several advantages, among which the possibility to cross the founder with any Cre tissue specific expressing lines and creating double transgenic animals expressing specifically SMO potentially in any tissue. In our genetic model system *JoSMOrec*, SMO activity resulted higher in the neocortex of young mice compared to controls, leading to a higher neuronal death rate during ageing in this brain region. In fact, no difference of SMO activity was detected between *JoSMOrec* 20-months-old mice and aged-matched controls. In line with that, the immunohistochemical analyses using the neuronal marker NeuN showed a significant neuronal loss in old *JoSMOrec* mice in respect to controls. Consistently, a relatively low number of SMO positive neurons was detected in old Tg mice. While no differences were observed in APAO and ODC enzymatic activities, SSAT showed increased activity in the neocortex of both young and old SMO Tg mice. This increase likely represents a cellular response to compensate SMO overexpression, possibly via Spd acetylation and export or/and acetyl-Spd oxidation by APAO. The SSAT response, that should be neuroprotective, however could not overcome the SMO overexpression-induced neurotoxicity. We could not also rule out that increased SSAT activity could be responsible of Spm acetylation and consequently SMO substrate subtraction. This is in agreement with the higher Spd and Put content in the neocortex showed by young Tg mice. The immunohistochemical analysis of neocortex in *JoSMOrec* mice using GFAP and Iba1 markers showed a significant astroglial and microglial activation in old Tg mice. In order to rule out any hypothetical side-effects due to random transgene integration and consequently gene disruption, we performed immunohystochemical analyses of neocortex from non-transgenic littermates (WT) using antibodies directed against SMO, NeuN, GFAP and Iba1. No different expression of the markers analyzed was observed between WT and Sg mice. This indicates that SMO overexpression is affecting Tg phenotype, leading to a more pronounced brain damage during ageing, possibly due to a SMO-derived overproduction of H_2_O_2_ when cellular antioxidant defences become less efficient in old mice. In fact, H_2_O_2_ production resulted greatly enhanced in the neocortex of Tg mice compared to Sg controls, strongly suggesting that Tg mice were suffering a higher cellular oxidative stress. In order to evaluate the role of SMO in an excitotoxic condition, young Tg and Sg mice were treated with KA and behavioural phenotype was analyzed observing and scoring the induced seizures activity according to the well defined scale of Schauwecker [30]. The KA treated *JoSMOrec* mice showed a more severe behavioural phenotype with respect to the KA treated Sg ones, suggesting that SMO overexpression is affecting glutamatergic transmission. After KA treatment Tg mice showed in the neocortex a considerable astrogliosis and a stronger microgliosis compared to Sg mice, evident markers of brain injury. Also TEM analysis confirmed this pronounced brain damage, since a higher number of neocortical neurons with abnormal morphological features, as cytoplasmic condensation and strong nuclear basophilia, were observed in KA-treated Tg mice compared to KA-treated Sg ones. These morphological neuronal alterations describe a poorly understood degenerative state that has been detected in various pathological conditions [51]–[53]. Since *JoSMOrec* mice overexpressing SMO were clearly more sensitive to excitotoxic insult, PA metabolism was investigated to understand how it could affect glutamatergic transmission in mice treated with KA. The KA treatment provoked an increase of SMO and SSAT transcript and a more evident increase of SMO, APAO and SSAT enzymatic activity in both Tg and Sg mice, leading to the conclusion that the whole PA catabolism was induced. The noticeable increase of SMO and SSAT enzymatic activity observed could be only partially explained by transcript induction after KA injection, post-transcriptional control would be responsible for the higher enzymatic activity observed. No significant differences were observed in both APAO and ODC transcript accumulation in all the samples analyzed from Tg and Sg mice treated with KA and vehicle. While analyses of ODC transcript and enzymatic activity are completely matching, we could not observe any *APAO* gene induction and also in this case the increase of enzymatic activity measured is due to a post-transcriptional regulation. As expected, the increase in enzymatic activity is more evident in Tg than in Sg mice, this might be explained by the concomitant SMO induction and overproduction in *JoSMOrec* mice.The effect of SMO overexpression is clearly visible by immunohistochemical analysis of the Tg neocortices where astroglia and a microglia activation, as well as abnormal neurons, occur. When comparing PA content between Tg and Sg mice, it can be noticed that Spd and Put levels are higher in Tg mice than Sg ones, while no difference can be observed in the Spm level. This is pointing out that among PAs, Spm is well buffered in its cellular content and confirming that its homeostasis is crucial for physiological cell life. Nevertheless, we observed an increase of Spd that can be explained with Spm oxidation by SMO, while the increase of Put can be due to the concerted action of SSAT and APAO according to their enzymatic reaction. After KA treatment of *JoSMOrec* mice, the PAs content is further altered, as consequential of the whole PA catabolism activation. In fact, we observed a general decrease of PAs, but a difference in Spd level between Tg and Sg mice is still noticeable, being higher in Tg mice. This difference in Spm/Spdratio could explain the higher sensitivity to KA treatment in Tg mice. It is well demonstrated that intracellular PAs can cause rectification of AMPA and KA receptors, acting as internal cell blockers of these receptor channels to prevent the flux of Na^+^ and Ca^2+^ as the membrane is depolarized during synaptic activity [16]. The magnitude of these effects will depend on the relative levels of Spm, Spd and Put [16]. Since we observed a decrease of Spm/Spd ratio in *JoSMOrec* mice, most probably the increase of Spd level compete for Spm binding to AMPA and KA receptors, enhancing the KA effect on both receptors. This explanation is in line with the treatment of organotypic hippocampal slice cultures with *N,N* ^1^-bis(2,3-butadienyl)-1,4-butanediamine (MDL72,527), a SMO inhibitor, that resulted in a considerable neuronal protection against KA-induced toxicity and significantly prevented neuronal death in ischemia and mechanical injury models [54]. In the work of Liu et al [54] has been reported that while the pre-treatment with a combination of MDL72,527 and cyclosporin A (a blocker of the formation of the mitochondrial permeability transition pore) provided additive neuronal protection, on the contrary a combination of MDL72,527 and EUK-134 (a specific scavenger of hydrogen peroxide [55], [56]) did not produce an additive neuronal protective effect on KA neuronal toxicity. These observations strongly suggest that SMO overexpression is only partially affecting the increase of apoptosis after KA treatment, while is the major source of H_2_O_2_ production which increases vulnerability to a KA-induced neuronal death. This is in line with the work of Pledgie et al [57] who demonstrated by knockdown experiments that SMO, and not APAO, is the primary source of cytotoxic H_2_O_2_ in polyamine analogue-treated human breast cancer cell lines. It is well known that an excessive release of excitatory neurotransmitters such as glutamate importantly contributes to neuronal damage in cerebral ischemia, epilepsy, Alzheimer's disease and other forms of dementia. This excitotoxic insult is frequently accompanied by excess calcium influx and followed by generation of ROS, resulting in intracellular membrane damage and triggering apoptotic pathways, ultimately leading to cell death [37]. In fact, since endogenous Spm can block and/or modulate GluRs, alteration of Spm levels in *JoSMOrec* mice due to SMO overexpression can produce changes in Ca^2+^ flux through GluRs. During KA treatment there is an excessive activation of AMPA and KA receptors producing excitotoxicity leading to a consequent NMDA receptors activation [58], that in turn may drive Spm release from synaptic vesicles to synaptic cleft [59], [60]. The resulting neuronal and glial uptake of Spm could increase the cytoplasmic Spm level which is not compartmentalized in synaptic vesicles and as available as substrate for SMO enzyme activity. It can be noticed that there is a decrease of Spm content in both Sg and Tg mice after KA treatment, but the higher SMO level in *JoSMOrec* mice leads to an higher H_2_O_2_ production, contributing to the observed phenotype. The generation of Spm oxidation products, such as H_2_O_2_ and 3-aminopropanal, which spontaneously converts in acrolein, together with direct effects of Spm on AMPA and KA receptors, are likely involved in ROS increase and ultimately to neuronal degeneration and death. Within this scenario, and hypothesizing that SMO enzyme is one of the most important H_2_O_2_ producers in the brain, the transgenic *JoSMOrec* mice characterized in this work could represent a useful genetic model for studying brain pathologies such as epilepsy, Alzheimer's disease and other forms of dementia.

## Supporting Information

Figure S1 **Immunohystochemical analysis of neocortex from Sg and WT mice.** Sagittal brain slices from WT and Sg mice were stained with antibodies directed against SMO, NeuN, GFAP and Iba1. Slides of neocortex from 12 months old mice were analyzed. Cell counting is expressed as number of positive cells per 0.24 mm^2^ area. Statistical analyses were carried out with the one-way ANOVA test. WT, wild-type mice; Sg, syngenic mice.
(TIF)
