# Supplementary figures and images for: A New Transgenic Mouse Model for Studying the Neurotoxicity of Spermine Oxidase Dosage in the Response to Excitotoxic Injury
Source: PLoS One. 2013 Jun 19;8(6):e64810. doi: 10.1371/journal.pone.0064810 (PMC3686797; doi:10.1371/journal.pone.0064810)

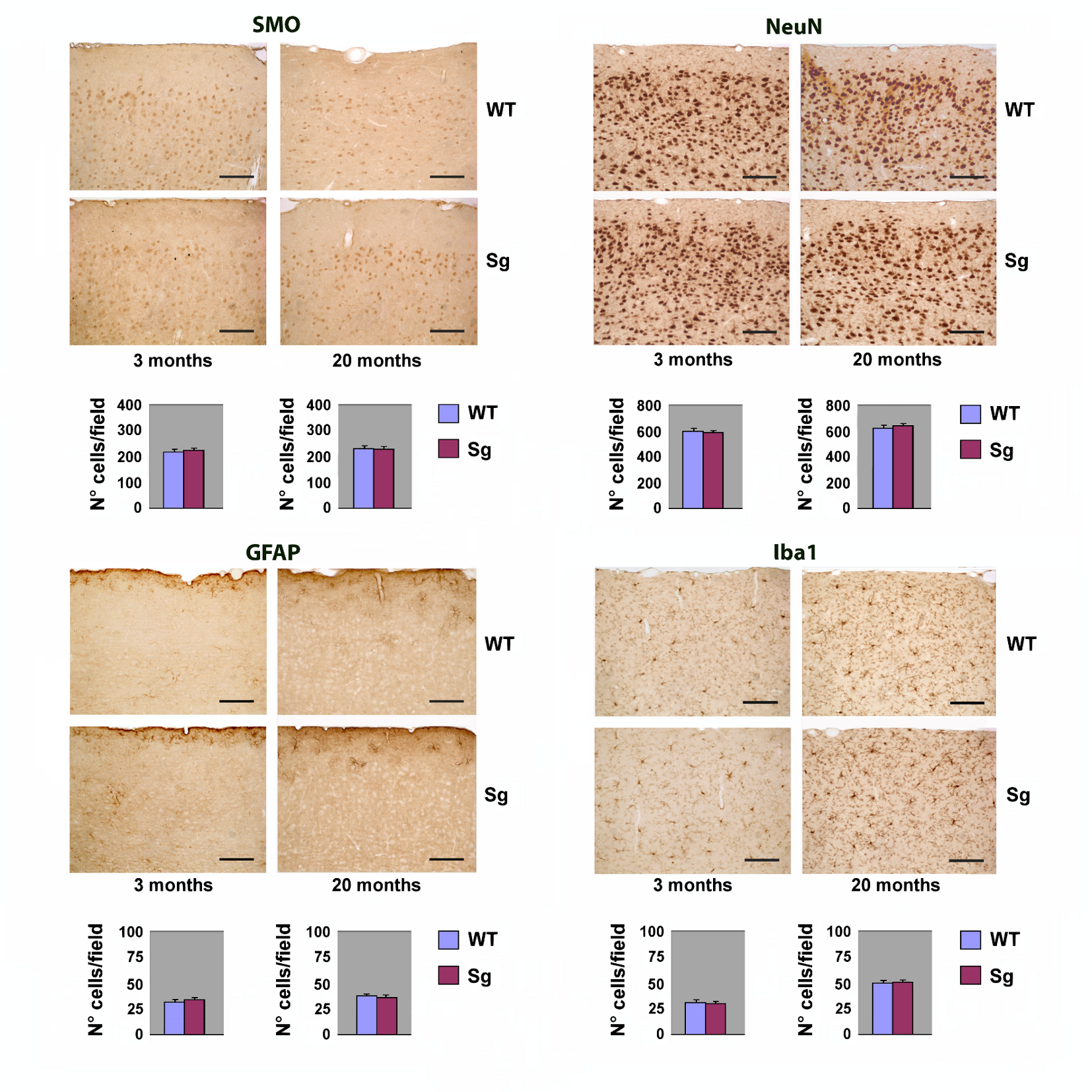

Supplement: Figure S1 — Immunohystochemical analysis of neocortex from Sg and WT mice. Sagittal brain slices from WT and Sg mice were stained with antibodies directed against SMO, NeuN, GFAP and Iba1. Slides of neocortex from 12 months old mice were analyzed. Cell counting is expressed as number of positive cells per 0.24 mm2 area. Statistical analyses were carried out with the one-way ANOVA test. WT, wild-type mice; Sg, syngenic mice. (TIF) [file pone.0064810.s001.tif]
